# Chemical and Physical Properties of the BLT4 Ultra Capacitor—A Suitable Material for Ultracapacitors

**DOI:** 10.3390/ma13030659

**Published:** 2020-02-02

**Authors:** B. Wodecka-Duś, M. Adamczyk-Habrajska, T. Goryczka, D. Bochenek

**Affiliations:** Faculty of Science and Technology, Institute of Materials Engineering, University of Silesia in Katowice, 75 Pułku Piechoty 1a, 41-500 Chorzów, Poland; beata.wodecka-dus@us.edu.pl (B.W.-D.); dariusz.bochenek@us.edu.pl (D.B.)

**Keywords:** lanthanum barium titanate (BLT4) ceramics, dielectric and electrical properties, posistor properties, piezoresistivity coefficients

## Abstract

This paper describes the properties of a lead-free ceramic material based on barium titanate, designed for the construction of ultracapacitors and sensors used in mechatronic systems. The admixture of lanthanum (La^3+^) served as a modifier. The ceramic powders were obtained by the solid phase reaction method (conventional method—mixed oxides method—MOM). Technological conditions of the synthesis process were determined on the basis of thermal analysis. The obtained samples are characterized, at room temperature (*T_r_* < *T_C_*), by a single-phase tetragonal structure and a P4mm space group. Properly developed large grains (*d* = 5 µm) contributed to the increase in electric permittivity, the maximum value of which is at the level of *ε_m_* ≈ 112,000, as well as to a strong decrease in specific resistance in the ferroelectric phase, whereas above the Curie temperature, by creating a potential barrier at their boundaries, there was a a rapid increase in resistivity. The temperature coefficient of resistance of the obtained posistor is 10.53%/K. The electrical properties of the obtained ceramics were examined using impedance spectroscopy. In order to analyze the obtained results, a method of comparing the behavior of the real object and its replacement system in a specific frequency region was used, whereas the Kramer–-Kroning (K–K) test was used to determine the consistency of the measured data. The proper selection of the stoichiometry and synthesis conditions resulted in the creation of an appropriate concentration of donor levels and oxygen gaps, which in turn resulted in a significant increase in the value of electrical permittivity, with small values of the angle of dielectric loss tangent. This fact predisposes the discussed material for certain applications (in the construction of ultracapacitors, among others).

## 1. Introduction

Barium titanate is a classic example of a perovskite ferroelectric. This material was discovered in the 1940s, but it is currently experiencing a kind of renaissance. BaTiO_3_ ceramic, not doped and not vacuum-heat treated, is a dielectric. Its resistance is *ρ* > 10^10^ Ω cm (for *T_r_* = 20 °C), and when properly doped, synthesized, and sintered, it can be a semiconductor with a resistance equal to *ρ* = (1 ÷ 10^2^) Ωcm (for *T_r_* = 20 °C) [1]. The strongest changes in conductivity are obtained for BaTiO_3_ doped with trivalent or pentivalent metals that enter the Ba or Ti subnetwork (Me^3+^ → Ba, Me^5+^ → Ti). In place of barium, the following ions may be substituted: Sb^3+^, Y^3+^, or lanthanide, and actinide ions (rare earth ions) Sm^3+^, La^3+^, Er^3+^, and Ho^3+^, and, in titanium ion positions, Ta^5+^ and Nb^5+^ ions. These metals are donor centers in BaTiO_3_. Small amounts of such admixtures (0.1 ÷ 0.3 at.%) cause a change in the conductivity of BaTiO_3_ by 8 ÷ 10 orders [2,3]. In addition, in the area of ferroelectric phase transition (~120 °C) there is a strong increase in resistivity (3 ÷ 6 orders)—The positive temperature coefficient of resistivity (PTCR) effect. In most cases, the introduction of lanthanide and actinide admixtures in BaTiO_3_ in excessive amounts or with the use of molar doping allows the procurement of semiconductive materials exhibiting a posistor effect [4].

Doped ceramic materials based on barium titanate, free from lead, undoubtedly have a wide range of application possibilities in the field of the so-called green, environmentally friendly technologies. They find practical applications in classic capacitors, thermistors and varistors [5,6,7,8], and are also used as components for the construction of multi-layer capacitors (multi-layer ceramic capacitor (MLCC)), piezoelectric sensors, and piezoelectric transducers [9,10,11]. Ceramic materials based on barium titanate are also used for the construction of ferroelectric random access memory (FeRAM) [12]. It is impossible not to also mention the integrated microelectronic-electro-mechanical systems (MEMS), in which appropriately modified ceramic material based on the BaTiO_3_ compound acts as a building material [13].

The literature on the subject reveals that barium titanate is a relatively easily modifiable compound, where one of the most effective additives is lanthanum [14,15,16,17]. Not without significance is its impact on increasing the specific resistance of BaTiO_3_, as well as a slight temperature dependence of electrical permittivity over a wide temperature range. Barium titanate and lanthanum oxide form solid solutions with each other and are described by the formula Ba_1−*x*_La*_x_*Ti_1−*x*/4_O_3_. The ions of La^3+^ lanthanum are introduced into subnet A of the ABO_3_ perovskite structure, in place of Ba^2+^ barium ions, acting as a donor dopant.

An additional positive charge is introduced into the sample, together with the lanthanum ions, which causes disruption of the electrical balance. The compensation of excess electrical charge (in order to preserve the electro-neutrality of the crystal lattice) can occur through the creation of cationic vacancies in the subnets A and B of the ABO_3_ structure (ion compensation) or by introducing an additional electron (electron compensation) [18]. Ion compensation gives a very weak effect and, at room temperature, the material is an insulator. In contrast, electronic compensation is the reason for the increase in conductivity, in which the number of charge carriers equals the charge of the lanthanum ion [16]. Lanthanum barium titanate is an alternative material to other lead-free perovskites such as K_0.5_Na_0.5_NbO_3_ [19], (K_0.38_Na_0.52_Li_0.04_)-(Nb_0.86_Ta_0.1_Sb_0.04_)O_2.97_ [20,21], and KNbO_3_ [21]. The BLT4 ceramics presented in the manuscript are characterized by a high value of dielectric permittivity at room temperature as well as at Curie temperature. Such high values of electric permittivity are not found for other representatives of the BLT system obtained by the classical method. The material in question can therefore be an attractive alternative in the construction of ultra-capacitors. Other properties (posistors application, among others) are very important for further application in sensors used in mechatronic systems.

The above-mentioned premises influenced the decision to undertake intensive research on barium titanate modified with lanthanum. As a result of the intensified activities described in earlier publications, ceramics with extremely interesting and previously unheard of application properties were obtained.

## 2. Sample Preparation

Lanthanum barium titanate (BLT4) was obtained by donor doping of pure BaTiO_3_ (BT) with La^3+^ lanthanum ions in amounts of 0.4 mol.%. The starting materials for obtaining BLT4 ceramics by the conventional (oxide) method were compounds for analysis: barium carbonate, BaCO_3_, lanthanum oxide, La_2_O_3_, and titanium (IV), TiO_2_—from Sigma–Aldrich. The substrates were weighed in a stoichiometric ratio, taking into account the expected chemical reaction:

0.996 BaCO_3_ + 0.002 La_2_O_3_ + 0.999 TiO_2_ → Ba_0.996_La_0.004_Ti_0.999_O_3_ + 0.996 CO_2_↑. (1)

In this way, BLT4 powders with the desired composition were obtained. The prepared material was dry mixed in a porcelain mortar for *t* = 1 h, and then subjected to wet milling with the addition of C_2_H_5_OH ethyl alcohol (99.99%, POCH), in a planetary ball mill for *t* = 24 h, using zirconium-yttrium balls as grinders.

The synthesis process was carried out at the temperature *T_s_* = 950 °C (during *t* = 2 h). The next phase of technological work was a three-stage sintering in an air atmosphere. The first two sinterings took place at *T*_I_ = 1250 °C and *T*_II_ = 1300 °C, respectively, for 2 h each. The third sintering process was carried out at three different temperatures. The obtained ceramic material was subjected to density, porosity, and water absorption tests (Table 1). Test results indicated that sintering at *T_3_* = 1350 °C best affects the basic parameters of ceramics.

## 3. Experimental Procedure

The crystalline structure of the investigated ceramics was studied using X-ray diffraction technique (XRD). The measurements were done at room temperature using an X’Pert Pro diffractometer (X‘Pert-Pro, Philips, Amsterdam, The Netherlands) equipped with CuKα_1_ and α_2_ radiation (40 kV, 30 mA). Diffraction patterns were measured in step-scan mode in the angular range from 10° to 90° 2θ, with a measuring step of 0.04° and a counting time of 10 s per step. Morphology of the BLT4 ceramics was observed with a scanning electron microscope (SEM) (Hitachi S-4700, Tokio, Japan).

The grain size measurements were performed on the fractured surface of the sintered ceramic samples, which were coated with gold to provide electrical conductivity and to avoid any charging effects. The temperature characteristics of dielectric permittivity and loss factor, as well as impedance data, were measured using an impedance analyzer (HP 4192A, Hewlett-Packard, Palo Alto, CA, USA) in the 20 Hz–2 MHz frequency range. The thermoresistive properties were investigated using a computerized automatic system based on a pikoamperometer (Keithley–6485, Cleveland, OH, USA). The samples intended for electrical measurements had the shape of discs with a diameter of 1 mm and a thickness of 0.5 mm. The properly cut and polished samples were coated with silver electrodes, using a silver paste.

## 4. Crystal Structure and Microstructure

**Figure 1:**
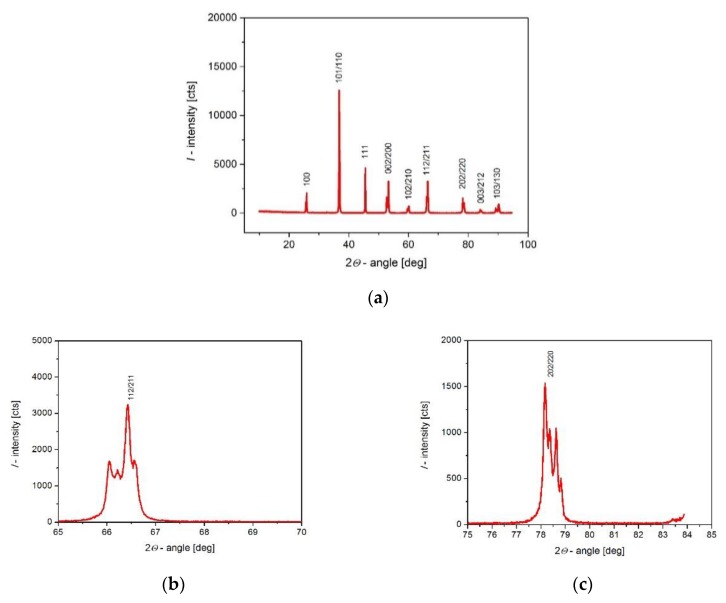
(**a**) X-ray diffraction pattern of BLT4 ceramics. (**b**) magnification of 112/221 peak; (**c**) magnification of 202/220 peak.

X-ray diffraction patterns of the investigated materials are presented in Figure 1. The detailed analysis showed that, at room temperature (*T_r_* < *T_C_*), the grains of the crystalline phase are single-phase, have a perovskite type structure with tetragonal symmetry (*a*_0_ = *b*_0_ ≠ *c*_0_, *α* = β = γ = 90°), have a primitive *P* Bravais unit cell, a 4mm point group, and a P4mm space group. Using the Rietveld method [22], the unit cell parameters were determined based on the X-ray spectra obtained: *a*_0_ = 0.3992 nm, *c*_0_ = 0.4020 nm, its volume, *V*, = 64.20 × 10^−30^ m^3^, and homogeneous tetragonal deformation parameter, δ*_T_* = 1.0070.

It should also be mentioned that a detailed analysis of diffraction reflections indicates that they are not excessively widened, which indicates low stresses in the crystal structure.

**Figure 2:**
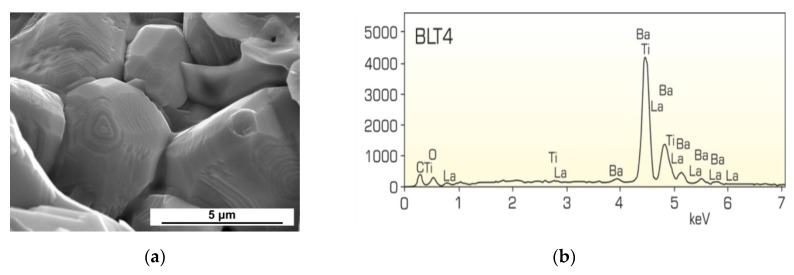
SEM images of fractures (**a**) and EDS emission spectrum (**b**) of BLT4 ceramics.

The tested BLT4 ceramic material was also subjected to microstructural analysis. Figure 2 presents SEM images of the microstructure for a magnification of ×10,000 together with X-ray microanalysis (EDS) emission spectra of the obtained BLT4 ceramics.

The appearance of the microstructure indicates that, during the fractures, some cracks occurred through the grains (transcrystalline cracks). The resulting ceramics are characterized by well-shaped large angular grains (*d* = 5 µm), with a tendency to spiral hexagonal growth. This two-dimensional grain growth mechanism significantly increases a single grain, and consequently increases the strength of the resulting ceramics. The qualitative results of the chemical composition analysis, carried out by the method of X-ray microanalysis, clearly indicate the chemical homogeneity of the samples and the absence of foreign admixtures and impurities. The quantitative analysis of the chemical composition was aimed at determining the degree of compliance of the actual element content with theoretical stoichiometry. The stoichiometric ratio of the starting components was converted into elements: oxygen (O), titanium (Ti), barium (Ba), and lanthanum (La). The difference between the theoretical and experimental chemical composition was ±2 wt.%, which is within the error limits of the method used (Table 2).

In summary, both point and surface EDS analyses performed for BLT4 ceramic samples confirmed the purity, as well as the experimentally assumed qualitative and quantitative composition, of the tested material.

## 5. Dielectric Properties

**Figure 3:**
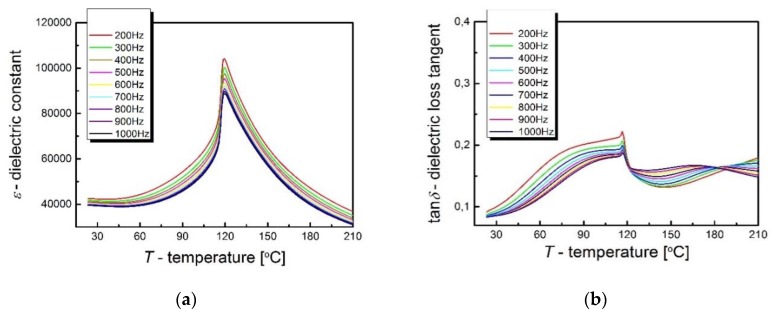
Temperature dependence of electric permittivity (**a**) and tangent of the dielectric loss angle (**b**) on the temperature and frequency of the measuring field for BLT4 ceramics.

The most important characteristic in the study of the dielectric properties of materials is the temperature dependence of electric permittivity (*ε*), which allows determination of the temperatures of phase transitions, as well as giving information about their type. In turn, the temperature characteristics of the tangent of the angle of dielectric loss (tan*δ*) allows us to draw conclusions about the loss of the tested material. The *ε* and tan*δ* temperature characteristics were measured at several selected frequencies of the measuring field, selected in the range *f* = (200 ÷ 1000) Hz, in the temperature range (20 ÷ 210) °C (Figure 3).

The obtained material shows a sharp phase transition, taking place at a temperature of *T_C_* = 120 °C and is characterized by a gigantic electrical permittivity value, which, at *T_C_* temperature, is *ε_max_* = 118,764 for the frequency of the measuring field *f* = 200 Hz. Electrical permittivity at room temperature reaches ~40,000 and is only slightly dependent on the frequency of the measuring field (∆*ε* = *ε*_200Hz_−*ε*_1000Hz_ = 42,409 − 39,497 = 2912). Frequency dispersion intensifies around the Curie temperature and is ∆*ε* = *ε*_200Hz_ − *ε*_1000Hz_ = 19,624, which constitutes 25% *ε_max_*_200Hz_. The values of dielectric permittivity at room temperature is very high in comparison with the other members of the BLT system. For example, the dielectric permittivity of Ba_0.08_La_0.02_TiO_3_ is smaller than 1000 [23]. Similar values of *ε* are characteristic for B_1−x_La_x_TiO_3_ (0.001 ≤ x ≤ 0.005) [24]. The sources of frequency dispersion should be seen in the increasing share of spatial charge in the sample volume and its polarization. It should be remembered that by admixing barium titanate with lanthanum, the trivalent La^3+^ lanthanum ions are substituted for the divalent Ba^2+^ barium ions, which results in an imbalance in the crystal lattice. In order to preserve the electrical neutrality of the unit cell, compensation mechanisms are activated, which are involved in creating *V_Ti_*, *V_Ba_*, *V_O_* vacancies. Literature reports indicate that, for low concentrations of lanthanum, neutrality of the crystal lattice is achieved by generating oxygen vacancies, which confirms the pale blue color of the obtained samples [11,24,25,26].

It is worth noting that the ceramics in question are characterized by low dielectric loss. At room temperature, the tangent of the loss angle is tan*δ_min_* ≈ 0.083, and at the phase transition temperature it is tan*δ_max_* ≈ 0.197. The low dielectric loss of the ceramic material in question is extremely important from the application point of view.

**Figure 4:**
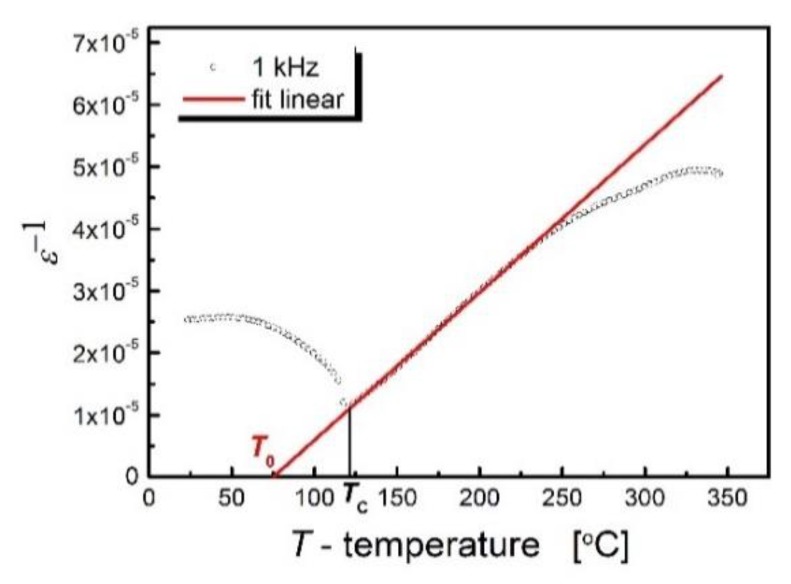
Inverse relative electrical permittivity from temperature for BLT4 ceramics.

A single sharp maximum appears on the *ε*(*T*) characteristics, while the 1/*ε*(*T*) relationship (Figure 4) satisfies the Curie–Weiss law in a wide range of temperatures of the paraelectric phase, starting from the Curie temperature. These facts indicate that the materials in question undergo a sharp phase transition.

Adjusting the above-mentioned Curie–Weiss law to the experimental data allowed us to determine the values of the Curie–Weiss temperature (*T*_0_ = 75 °C) and Curie constant (*C* = 4.20 × 10^5^ °C). In the examined ceramics, the Curie–Weiss temperature is located below the Curie temperature, which is a characteristic feature of ferroelectrics with a sharp phase transition and is characterized by a slight transformation blur. A summary of the basic dielectric parameters of BLT4 ceramics obtained at a measurement field frequency of *f* = 1 kHz is presented in Table 3.

## 6. Electrical Properties

**Figure 5:**
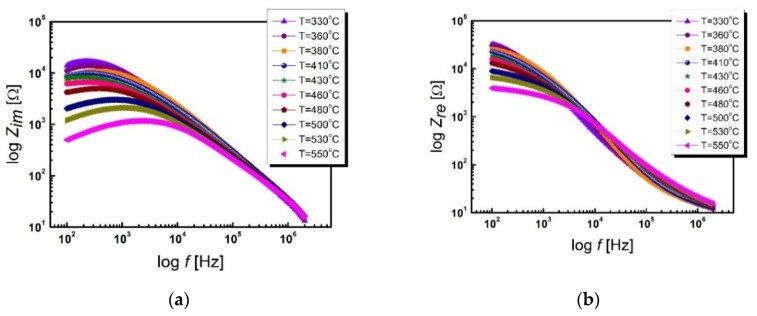
Frequency dependence of the impedance of the imaginary part, *Z_im_*, (**a**) and the real part, *Z_re_*, (**b**) on the frequency, *f*, at different temperatures of BLT4 ceramics.

The electrical conductivity of ceramics is closely related to the relaxation processes that take place inside it, i.e., in the areas of grains, intergranular boundaries, and electrode areas [27]. The question arises as to what particular role lanthanum plays in creating the conductivity of grains and grain boundaries. An in-depth analysis of the obtained results of impedance spectroscopy measurements will provide the answer to this question. Figure 5 shows the impedance spectra as a function of frequency obtained for several temperatures in the 330–550 °C range.

**Figure 6:**
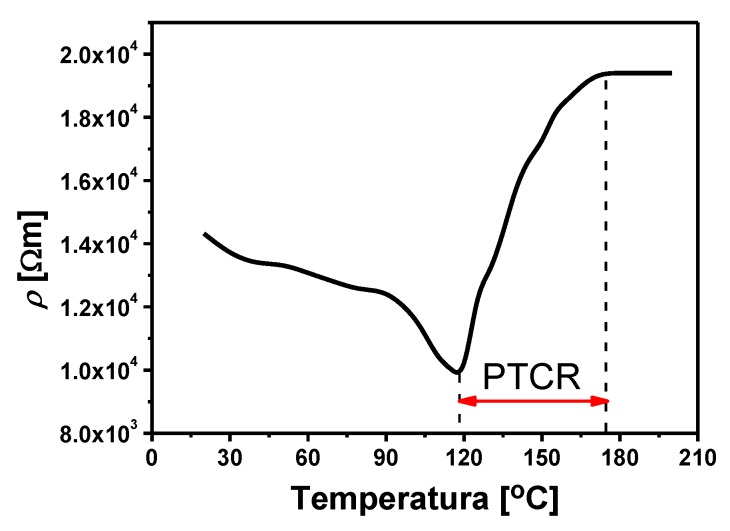
Residual spectrum (residuals) showing the frequency relationship of the relative difference between the experimental data and the data obtained as a result of the Kramers–Kroning (K-K) test at *T* = 230 °C for BLT4 ceramics.

The measuring points, representing the impedance experimental data, are arranged on smooth curves, which allow us to presume the correctness of the measurement. In order to obtain complete certainty as to the consistency of the measurement data, a test was carried out based on the Kramers–Kroning (K–K) equations [28,29,30]. Sample residual spectra showing the frequency relationship of the relative difference between the experimental data and the data obtained as a result of the K–K test for BLT4 ceramics at a temperature of *T* = 230 °C are shown in Figure 6.

The value of residuals did not exceed 0.5%, and their distribution relative to the frequency axis is random, which clearly suggests that the obtained impedance data are consistent and consistent with the assumed model. They can therefore be subjected to further detailed analysis.

One of the characteristics of *Z_im_*(*f*) of BLT4 ceramics, in the studied temperature range, appears in the low frequency range (10 ÷ 1000) Hz, well outlined local maximum, whose position on the frequency axis shifts to higher values with increasing measurement temperature. The observed changes are more of a change in the slope of the curves than a sharp maximum. In contrast, the relationship, logZ*_re_*(*f*), is characterized by rapid changes in almost the entire frequency range studied. In addition, in the case of this ceramic, there are no noticeable large temperature changes in log*Z_re_*(*f*) values—it can be said that the characteristics, starting from a frequency of 100 Hz, almost combine into one run for all temperatures tested. Most likely, this behavior is caused by the release of the spatial charge as a result of a change in the potential barrier [31,32].

The next stage in the interpretation of the received impedance spectra was the selection of an electric replacement system that fully describes the phenomena occurring inside the ceramic material.

**Figure 7:**
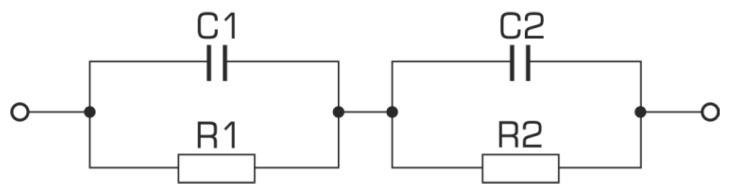
Electric equivalent RC system.

It is known that ionic or mixed conductivity occurs in compounds of the perovskite type structure; both grains and grain boundaries, as well as electrode areas, contribute to the impedance value [33,34]. The complexity of the occurring phenomena makes finding an appropriate replacement system that correctly represents the individual components of the sample difficult. An additional nuisance is the fact that it is possible to find more than one replacement system that correctly simulates the measured electrical response. So the question arises of which of the systems is a correct reflection of the properties of the electrically active areas of the sample that are connected to each other. Presentation of the electrical response of the system on the complex plane obtained as a result of measurements of the so-called Nyquist charts make it much easier to choose the right layout. In an ideal situation, to describe this type of impedance spectra, a substitute system is used, consisting of serially connected RC elements, representing a lossy capacitor, and consisting of capacitors (*C*) and resistance (*R*) connected in parallel (Figure 7).

**Figure 8:**
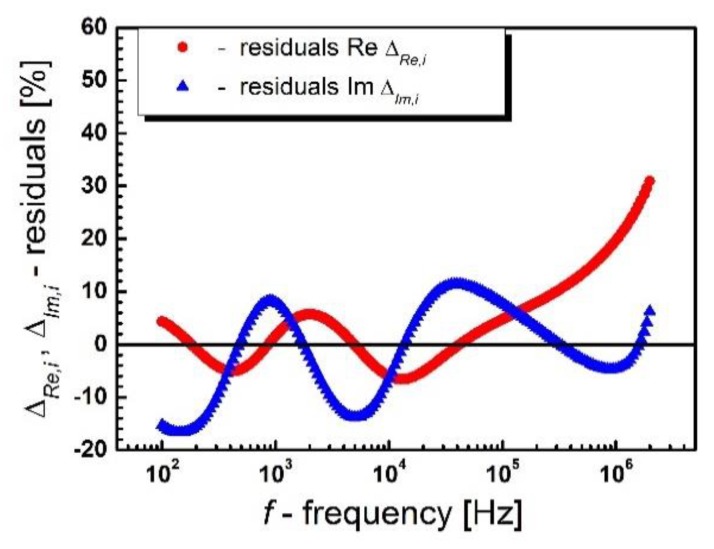
Frequency relationship of approximation errors of the real part and the imaginary impedance of BLT 4 ceramics, calculated at *T* = 377 °C.

The attempts undertaken to match the discussed system to experimental data did not bring a satisfactory result, which was clearly indicated by the large values of approximation errors of the real and imaginary impedances placed on the fit compatibility graph (Figure 8).

**Figure 9:**
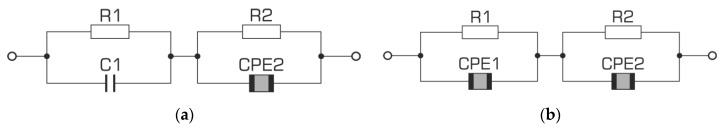
RC electric equivalent systems with fixed constant-phase elements CPE: With single element CPE (**a**) and with double element CPE (**b**).

Consequently, the replacement system had to be modified. Based on the literature data on impedance spectrum analysis [35], two circuits were proposed for further testing, slightly different from the original system. The difference was the replacement of one or two capacitive elements with constant-phase elements CPE (Figure 9).

The proposed solutions are often used in the analysis of the electrical response of solids [36]. The use of CPE elements is a common practice, not only in the case of testing the electrical properties of systems based on lanthanum barium titanate [36,37], but also other compounds with crystal structures significantly differing from classic perovskites [38,39].

**Figure 10:**
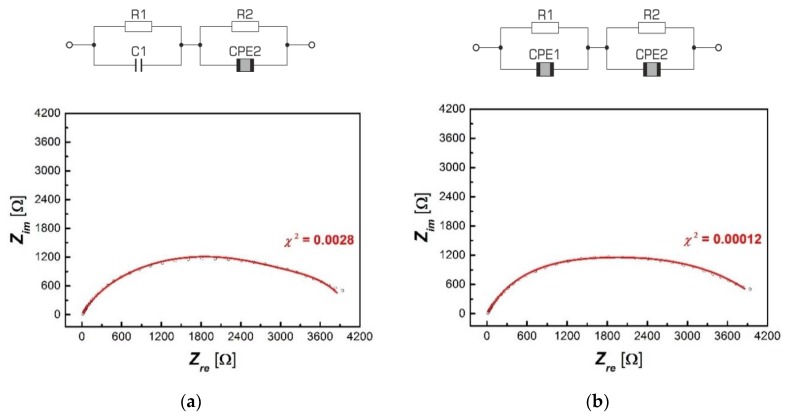
Graphs of the dependence of the imaginary part of impedance (*Z_im_*) on its real part (*Z_re_*) together with the matching spectrum for selected electric replacement models obtained at *T* = 455 °C; for a system with a single CPE (**a**) and for a system with a double CPE (**b**).

The discussed modification significantly improved the quality of the fittings obtained for BLT4 ceramics, which is visible in Figure 10, with much better results when using the double CPE element (Figure 10b), which confirms the minimal discrepancy between the measuring points and the matching curves and a smaller value of the numerical parameter matches *χ*^2^.

**Figure 11:**
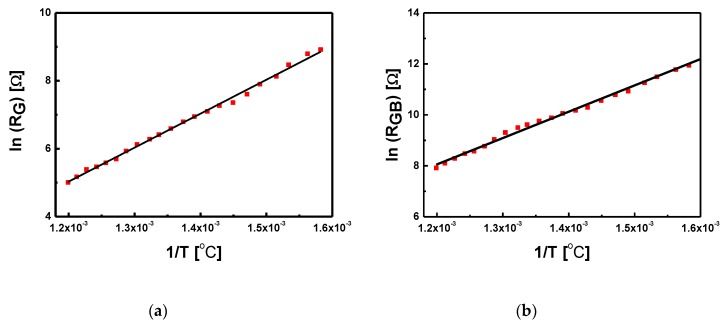
Dependence of the natural logarithm of the grain (**a**) and grain boundary (**b**) values calculated on the basis of impedance spectra as a function of temperature inverse of BLT4 ceramics.

The characteristics of *Z_im_*(*Z_re_*) take the form of deformed, at times asymmetrical, single semi-circles, and their shape allows us to conclude that the obtained relationships are the result of the imposition of two semi-circles representing the electrical response of grains and grain boundaries. The selected replacement circuits (consisting of two serially connected RC elements, modified with one or two constant phase CPE elements) were used to analyze the impedance data obtained for BLT4 ceramic samples. As a result of the adjustments made, the temperature dependence of the individual parameters of the above-mentioned replacement systems was determined, in particular the resistivity of both RC elements, which—as already mentioned—can be interpreted as the resistivity of grains and grain boundaries. The obtained results indicate that the resistivity of grain boundaries is much higher (*R_GB_* = 2741 Ω) than the resistivity of the grains themselves (*R_G_* = 149 Ω). For each case, the relation ln*R_G_*(1/*T*) and ln*R_GB_*(1/*T*) was plotted—Figure 11.

The presented relationships can be described by a linear function, which indicates the activation nature of the process and, thus, can be described using the Arrhenius Formula (2):

*R* = *R*_0_exp(−*E_a_*/*kT*) .X (2)

Based on the expression (2), activation energy of conductivity processes in grains, *E_G_* = 0.87 ± 0.02 eV, and grain boundaries, *E_GB_* = 0.89 ± 0.01 eV, was determined. The difference between the activation energy value of grains and inter-grain boundaries is small, which indicates no reduction in ion mobility within the grain boundaries.

## 7. Thermoresistive Properties

**Figure 12:**
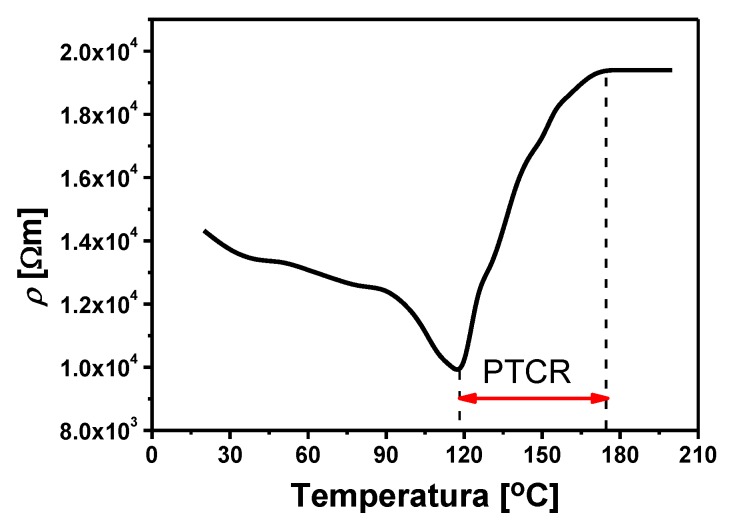
Temperature dependence of resistivity for BLT4 ceramics.

The results of tests on direct current electrical conductivity of BLT4 ceramics, obtained at a constant voltage of *U* = 10 V in the temperature range (20 ÷ 200) °C, are shown in Figure 12, in the form of a specific resistivity (*ρ*) depending on the temperature (*T*). In the temperature range 120 °C–170 °C the PTCR effect is visible.

An important factor in the description of the PTCR effect is the value of the temperature resistance coefficient, *α_T_*, which is defined as the relative change in resistance related to temperature change and can be calculated for each point on the *R*(*T*) curve:

(3) $$\alpha_{T}=\frac{1}{R}\cdot \frac{dR}{dT}=\frac{d\ln R}{dT}=\ln10\cdot \frac{d\mathrm{lg}R}{dT}$$

In the range of resistance jump between *R_PTC_*_min_ and *R_PTC_*_max_, the *α_T_* factor can be considered approximately constant, which causes the relationship (4) to take the following form:

(4) $$\alpha_{T}=\frac{\ln\left(\frac{R_{2}}{R_{1}}\right)}{T_{2}-T_{1}}$$

Additional posistor parameters, in addition to the basic dependence of the resistance on the temperature and the temperature coefficient of resistance (*α_T_*), are also the values of resistance at different temperatures (*ρ_min_* and *ρ_max_*), the temperature of the beginning of resistance increase (*T_PTCRmin_*), and the maximum temperature of resistance (*T_PTCRmax_*), thermal-time constants and voltage dependencies, dissipated power factor, and heat capacity of the element [2]. Based on the course of the *R*(*T*) function and Formula (4), the basic posistor parameters of BLT4 ceramics were calculated. The obtained values are summarized in Table 4.

The analysis of the above results clearly shows that BaTiO_3_ ceramic containing 0.4 mol.% lanthanum has properties characteristic for posistors and has a positive temperature coefficient (*α_T_* = 10.53%/K) in the temperature range (120 ÷ 170) °C. For comparison, the leading lead-free posistor ceramic based on barium titanate has a *α_T_* factor of 21.4%/K [40], and, in the best ceramic posistor, the parameter discussed can have values up to 30%/K [41]. The wide temperature range of the PTCR effect in the obtained materials also deserves to be emphasized.

## 8. Conclusions

The results of X-ray examinations clearly indicate that, as a result of the technology used, a single-phase material was obtained exhibiting a perovskite-type structure with tetragonal symmetry at room temperature. The appropriate selection of dopant concentration, as well as the technological conditions, resulted in obtaining ceramics characterized by well-shaped large angular grains (*d* = 5 µm), with a tendency to spiral hexagonal growth. Such shaped grains affect the high quality of ceramics, increasing their mechanical strength. The proper selection of the synthesis conditions resulted in the creation of an appropriate concentration of donor levels and oxygen gaps, which in turn resulted in a significant increase in the value of electrical permittivity, with small values of the angle of dielectric loss tangent. This fact predisposes the discussed material for certain applications (in the construction of ultracapacitors, among others). The appropriate level of material defect also affected the lack of reduction of ion mobility within the grain boundaries and the appearance of posistor properties in the temperature range of 120 ÷ 170 °C.

An additional advantage of the material obtained is that it is an economic and environmentally friendly technology. The discussed ceramic material could be successfully used in innovative electronic components dedicated to applications in modern mechatronic and automatic systems.

## Figures and Tables

**Table 1:** Comparison of actual density (ρexp), water absorption (N), and porosity (P) of lanthanum barium titanate (BLT4) ceramics depending on the third sintering temperature (TIII).

Parameter	Values		
*T*_III_ [°C]	1325	1350	1400
*ρ_exp_* [kg/m^3^]	5173	5633	5062
*P* [%]	4.7	2.4	6.6
*N* [%]	0.82	0.52	1.35

**Table 2:** Theoretical and experimental percentages of BLT4 ceramic elements.

Element	Marked Content of Elements from EDS [wt.%]	Theoretical Content of Elements [wt.%]	Difference of Determined Value in Relation to the Theoretical [wt.%]
O	20.0	20.6	0.6
Ti	19.3	20.5	1.2
Ba	60.6	58.6	2.0
La	0.1	0.2	0.1

**Table 3:** Values of dielectric parameters of BLT4 ceramics for the frequency of the measuring field f = 1 kHz.

Parameter	*T_C_* [°C]	*T*_0_ [°C]	*C* [°C]	*ε* in *T_r_*	*ε_max_*	tan*δ* in *T_r_*	tan*δ* in *T_C_*
BLT4	120	75	4.20 × 10^5^	39,497	99,140	0.083	0.197

**Table 4:** Values of posistor parameters of BLT4 ceramics.

Parameter	*T_C_* [°C]	*ρ_min_* [Ω∙m]	*ρ_max_* [Ω∙m]	Δ*T_PTCR_* [°C]	*α_T_* [%/K]
BLT4	120	9.71 × 10^3^	1.94 × 10^4^	120–170	10.53

## References

[1] Xu Y. (1991). Ferroelectric Materials and their Applications.

[2] Hozer L. (1990). Półprzewodnikowe Materiały Ceramiczne z Aktywnymi Granicami Ziarn.

[3] Mukherjee N., Roseman R. (2002). Microstructural Dependence of the Voltage Sensitivity of the PTCR Effect in Donor Doped Barium Titanate. Ferroelectrics.

[4] Busca G., Buscaglia V., Leoni M., Nanni P. (1994). Solid-state and surface spectroscopic characterization of BaTiO_3_ fine powders. Chem. Mater..

[5] Ianculescu A., Mocanu Z.V., Curecheriu L.P., Mitoseriu L., Padurariu L., Trusca R. (2011). Dielectric and tunability properties of La-doped BaTiO_3_ ceramics. J. Alloys Compd..

[6] Vijatović Petrović M.M., Bobić J.D., Grigalaitis R., Stojanović B.D., Banys J. (2013). La-doped and La/Mn-co-doped barium titanate ceramics. Acta Phys. Pol. A.

[7] Wodecka-Dus B., Plonska M., Czekaj D. (2013). Synthesis, microstructure and the crystalline structure of the barium titanate ceramics doped with lanthanum. Arch. Metall. Mater..

[8] Morrison F.D., Sinclair D.C., West A.R. (1977). Principles and Applications of Ferroelectrics and Related Materials.

[9] Xu J.B., Zhai J.W., Yao X. (2009). Structure and dielectric nonlinear characteristics of BaTiO_3_ thin films prepared by low temperature process. J. Alloys Compd..

[10] Morrison F.D., Sinclair D.C., Skakle J.M., West A.R. (1998). Novel doping mechanism for very-high-permittivity barium titanate ceramics. J. Am. Ceram. Soc..

[11] Morrison F.D., Sinclair D.C., West A.R. (1999). Electrical and structural characteristics of lanthanum-doped barium titanate ceramics. J. Appl. Phys..

[12] Agneni A., Paolozzi A., Sgubini S. (2001). Piezoceramic devices modeled as mechanical systems in finite element codes. COST.

[13] Makino H., Asai M., Tajima S., Kamiya N. (2002). Piezoresistive ceramic composite for the miniature force-sensor. Commun. Res. Rep..

[14] Puli V.S., Li P., Adireddy S., Chrisey D.B. (2015). Crystal structure, dielectric, ferroelectric and energy storage properties of La-doped BaTiO_3_ semiconducting ceramics. J. Adv. Dielectr..

[15] Caia W., Fu C., Lin Z., Deng X., Jiang W. (2012). Influence of Lanthanum on Microstructure and Dielectric Properties of Barium Titanate Ceramics by Solid State Reaction. Adv. Mater. Res..

[16] Morrison F.D., Coats A.M., Sinclair D.C., West A.R. (2001). Charge Compensation Mechanisms in La-Doped BaTiO_3_. J. Electroceram..

[17] Urek S., Drofenik M. (1999). PTCR Behaviour of Highly Donor Doped BaTiO_3_. J. Eur. Ceram. Soc..

[18] Wodecka-Duś B., Lisińska-Czekaj A., Czekaj D. (2012). Influence of lanthanum concentration on properties of BLT electroceramics. Key Eng. Mater..

[19] Adamczyk-Habrajska M., Szafraniak-Wiza I., Goryczka T., Szalbot D. (2020). Impedance Studiem of K_0.5_Na_0.5_NbO_3_ ceramics prepared from mechanochemically activated powders. Mater. Chem. Phys..

[20] Slodczyk A., Gouadec G., Colomban P., Pham-Thi M. Stress-modified phase transitions in polarized PMN-PIN-PT, KN and KNL-NTS single crystals/textured ceramics: Thermal expansion and Raman scattering studies. Proceedings of the 2013 Joint IEEE International Symposium on Applications of Ferroelectric and Workshop on Piezoresponse Force Microscopy (ISAF/PFM).

[21] Gouadec G., Colomban P., Slodczyk A., Pham-Thi M. Stress and temperature driven phase transitions in single crystalline KNbO3 and textured KNL-NTS ceramics: A Raman and thermal expansion study. Proceedings of the 2014 Joint IEEE International Symposium on the Applications of Ferroelectric, International Workshop on Acoustic Transduction Materials and Devices & Workshop on Piezoresponse Force Microscopy.

[22] Young R.A. (1995). The Rietveld Method.

[23] Ganguly M., Rout S.K., Sinha T.P., Sharma S.K., Park H.Y., Ahn C.W., Kim I.W. (2013). Characterization and Rietveld Refinement of A-site deficient Lanthanum doped Barium Titanate. J. Alloys Compd..

[24] Kuwabara M., Matsuda H., Kurata N., Matsuyama E. (1997). Shift of the Curie point of barium titanate ceramics with sintering temperature. J. Am. Ceram. Soc..

[25] Vijatović Petrović M.M., Bobić J.D., Ramoska T., Banys J., Stojanović B.D. (2011). Electrical properties of lanthanum doped barium titanate ceramics. Mater. Charact..

[26] Devi S., Jha A.K. (2009). Structural, dielectric and ferroelectric properties of tungsten substituted barium titanate ceramics. Asian J. Chem..

[27] Abrantes J.C.C., Labrincha J.A., Frade J.R. (2000). An alternative representation of impedance spectra of ceramics. Mater. Res. Bull..

[28] Boukamp B.A. (2004). Electrochemical impedance spectroscopy in solid state ionics, recent advances. Solid State Ion..

[29] Boukamp B.A. (1995). A linear Kronig–Kramers transform test for immittance data validation. J. Electrochem. Soc..

[30] Boukamp B.A. (1986). A nonlinear least squares fit procedure for analysis of immittance data of electrochemical systems. Solid State Ion..

[31] Kumar A., Mumari N.M., Katiyar R.S. (2009). Investigation of dielectric and electrical behavior in Pb(Fe_0.66_W_0.33_)_0.50_Ti_0.50_O_3_ thin films by impedance spectroscopy. J. Alloys Compd..

[32] Kumari L.K., Prasad K., Choudhary R.N.P. (2008). Impedance spectroscopy of (Na_0.5_Bi_0.5_)(Zr_0.25_Ti_0.75_)O_3_ lead-free ceramic. J. Alloys Compd..

[33] Nocuń M. (2003). Wprowadzenie do Spektroskopii Impedancyjnej w Badaniach Materiałów Ceramicznych.

[34] Bauerle J.E. (1969). Study of solid electrolyte polarization by a complex admittance method. J. Phys. Chem. Solids.

[35] Mancić D., Paunović V., Vijatović M., Stojanović B., Zivković L. (2008). Electrical characterization and impedance response of lanthanum doped barium titanate ceramics. Sci. Sinter..

[36] Lasia A. (1999). Electrochemical Impedance Spectroscopy and It’s Applications. Modern Aspects of Electrochemistry.

[37] Mancić D., Paunović V., Petrusić Z., Radmanović M., Zivković L. (2009). Application of impedance spectroscopy for electrical characterization of ceramics materials. Electronics.

[38] Kathayat K., Panigrahi A., Pandey A., Kar S. (2012). Characterization of electrical behavior of Ba_5_HoTi_3_V_7_O_30_ ceramic using impedance analysis. J. Mater. Sci. Appl..

[39] Parida B.N., Das P.R., Padhee R., Choudhary R.N.P. (2012). Synthesis and characterization of a tungsten bronze ferroelectric oxide. Adv. Mater. Lett..

[40] Shimada T., Touji K., Katsuyama Y., Takeda H., Shiosaki T. (2007). Lead free PTCR ceramics and its electrical properties. J. Eur. Ceram. Soc..

[41] Kainz G. (1998). Ceramic PTC Thermistors for Protection of Electronic Circuits.

