# Correction: Wodecka-Dus, B. et al., Chemical and Physical Properties of the BLT4 Ultra Capacitor—A Suitable Material for Ultracapacitors *Materials* 2020, *13*, 659

**DOI:** 10.3390/ma13051261

**Published:** 2020-03-10

**Authors:** Beata Wodecka-Dus, Malgorzata Adamczyk-Habrajska, Tomasz Goryczka, Dariusz Bochenek

**Affiliations:** Faculty of Science and Technology, Institute of Materials Engineering, University of Silesia in Katowice, 75 Pułku Piechoty 1a, 41-500 Chorzów, Poland; beata.wodecka-dus@us.edu.pl (B.W.-D.); tomasz.goryczka@us.edu.pl (T.G.); dariusz.bochenek@us.edu.pl (D.B.)

The authors wish to make the following corrections to this paper [1]:

The manuscript contains one mistake, namely, Figure 6 is incorrect.

The correct Figure 6 is as follows:

The authors would like to apologize for any inconvenience caused to the readers by this change.

## Figures and Tables

**Figure 6:**
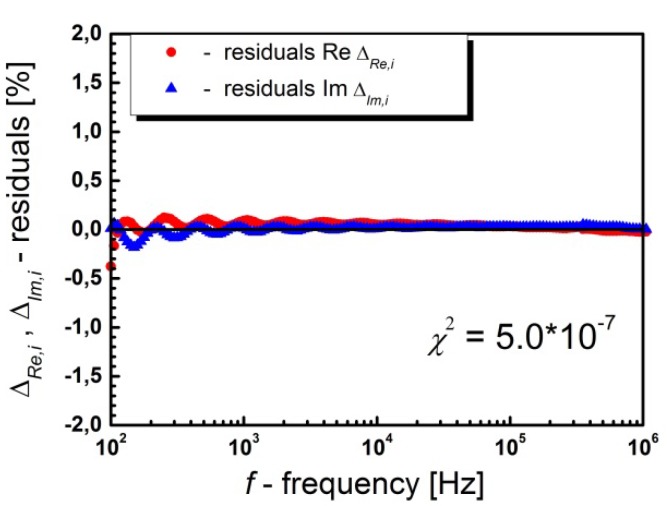
Residual spectrum (residuals) showing the frequency relationship of the relative difference between the experimental data and the data obtained as a result of the K-K test at *T* = 230 °C for BLT4 ceramics.
